# Effects of Aging on Postural Responses to Visual Perturbations During Fast Pointing

**DOI:** 10.3389/fnagi.2018.00401

**Published:** 2018-12-04

**Authors:** Yajie Zhang, Eli Brenner, Jacques Duysens, Sabine Verschueren, Jeroen B. J. Smeets

**Affiliations:** ^1^Department of Human Movement Sciences, Vrije Universiteit Amsterdam, Amsterdam Movement Sciences, Amsterdam, Netherlands; ^2^Department of Rehabilitation Sciences, FaBer, KU Leuven, Leuven, Belgium; ^3^Department of Kinesiology, FaBer, KU Leuven, Leuven, Belgium

**Keywords:** postural control, goal-directed, reaching, visual information, target, background, elderly, adjustment

## Abstract

People can quickly adjust their goal-directed hand movements to an unexpected visual perturbation (a target jump or background motion). Does this ability decrease with age? We examined how aging affects both the timing and vigor of fast manual and postural adjustments to visual perturbations. Young and older adults stood in front of a horizontal screen. They were instructed to tap on targets presented on the screen as quickly and accurately as possible by moving their hand in the sagittal direction. In some trials, the target or the background moved laterally when the hand started to move. The young and older adults tapped equally accurately, but older adults’ movement times were about 160 ms longer. The manual responses were similar for the young and older adults, but the older adults took about 15 ms longer to respond to both kinds of visual perturbations. The manual responses were also less vigorous for the older adults. In contrast to the young adults, the older adults responded more strongly to the motion of the background than to the target jump, probably because the elderly rely more on visual information for their posture. Thus, aging delays responses to visual perturbations, while at the same time making people rely more on the visual surrounding to adjust goal-directed movements.

## Introduction

Reaching out for objects while standing happens often in many daily life situations, such as when preparing a meal. In such situations it is essential to account for the forces that accompany reaching out so that they do not disturb one’s balance. This is achieved through anticipatory postural adjustments (Bouisset and Zattara, 1987; Massion and Dufosse, 1988; Aruin and Latash, 1995). Maintaining balance is not only essential because one does not want to fall, but also because allowing balance to be disturbed will challenge the accuracy of the endpoint of the reaching movement (Berrigan et al., 2006). As one gets older, maintaining balance when reaching forward while standing becomes more difficult (Hageman et al., 1995). Do such effects of aging influence the control of goal-directed movements?

People rely on continuously updated sensory information to rapidly adjust goal-directed movements (Cluff et al., 2015; Smeets et al., 2016). Such information comes from vision (Franklin and Wolpert, 2008; Oostwoud Wijdenes et al., 2013), the vestibular system (Keyser et al., 2017) and the somatosensory system (Lowrey et al., 2017). The adjustments’ latencies depend on the kind of sensory input. The arm takes between 100 ms and 160 ms to respond to a visually perceived target jump (Brenner and Smeets, 1997; Gritsenko et al., 2009; Oostwoud Wijdenes et al., 2013; reviewed by Smeets et al., 2016) or background motion (Brenner and Smeets, 1997; Whitney et al., 2003; Gomi et al., 2006). Even when adjusting reaching movements in response to such visual perturbations, postural responses can precede the hand’s response (Zhang et al., 2018). Does this ability to adjust movements decrease with age? The problems in balance control that develop during aging, combined with weaker muscles (Doherty, 2003) and poorer visual sensitivity and processing speed (Fiorentini et al., 1996; Owsley, 2011; Habekost et al., 2013) suggest that responses might become less vigorous and have longer latencies, both for target jumps and background motion.

Little is known about how aging affects the vigor of responses. Aging could reduce vigor because the muscles become weaker (Goodpaster et al., 2006) due to an age-related loss of spinal motor neurons and motor units, which reduces muscle fiber number and cross-sectional area (Booth et al., 1994). However, it has been reported that, older adults move less vigorously, irrespective of task difficulty in Fitts’ Task (Temprado et al., 2013). Therefore, the vigor of hand responses might be constrained by processing the information of the ongoing hand movement rather than by muscle strength. For postural responses, it is relevant that aging is associated with a reduced sensitivity of the proprioceptive (Skinner et al., 1984) and vestibular systems (Anson and Jeka, 2016). Therefore, we expect that older adults will rely more on vision of their surrounding when performing goal-directed movements (Coats and Wann, 2011; Chancel et al., 2018), and thus possibly show more vigorous manual responses to background motion, because manual responses to background motion may also be corrections for assumed self-motion (Gomi, 2008). Therefore, it is interesting to investigate the effect of aging on the timing and vigor of various responses to visual perturbations and to determine whether the effects are related to the general slowing of the movement.

Aging has been reported to delay the onset of fast responses to sudden visual perturbations: hand movement adjustments to target jumps and to background motion take about 20 ms longer in older adults (Kadota and Gomi, 2010; Kimura et al., 2015). It has been argued that these reflexive adjustments are essential for guiding the hand accurately to its target (Scott, 2016; Smeets et al., 2016), so a delayed response in older adults would decrease their accuracy. Additionally, larger postural sway in older adults when standing (Baloh et al., 1994; Blaszczyk et al., 1994; Laughton et al., 2003) may affect the accuracy of the endpoint of the reaching movement (Berrigan et al., 2006). A way to compensate for this reduced accuracy is by increasing the movement duration. There is indeed evidence that older adults move more slowly to maintain accuracy (Goggin and Meeuwsen, 1992; Temprado et al., 2013). We therefore test whether the longer adjustment latencies are related to longer movement times with increasing age.

In this study, we apply lateral visual perturbations (either target jump or background motion) while standing participants make forward reaching movements. The aim of the study is to investigate the effects of aging on responses to such sudden visual perturbations during an on-going reaching movement. The perturbations evoke responses in the goal-directed arm movements, so participants need to adjust their posture as well. We therefore also examine adjustments to the head and trunk.

## Materials and Methods

### Participants

Sixteen young adults (28 ± 3 years, seven males) and 16 older adults (74 ± 4 years, nine males) participated in this study. They were all right-handed, had normal or corrected-to-normal vision, and had no disease that is known to affect motor or sensory function. The study was approved by the Research Ethics Committee of the Faculty of Behavioral and Movement Sciences, Vrije Universiteit Amsterdam (no. VCWE-2016-176R1). Written informed consent was obtained from each participant.

### Experimental Setup and Procedure

**Figure 1:**
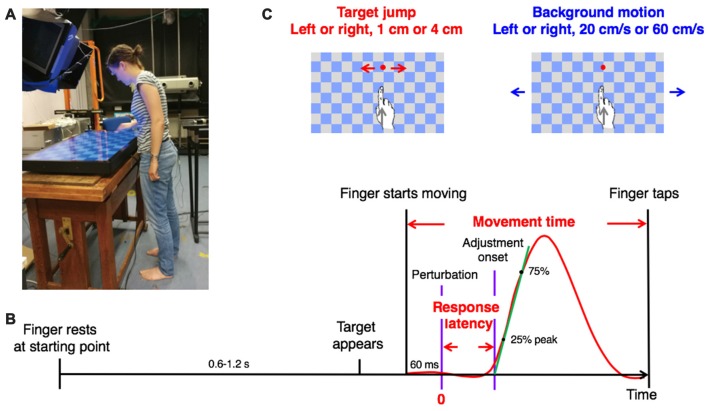
Methods. **(A)** A young participant making a movement in the experimental setup. Usage of image is with written informed consent. **(B)** Sequence of events in a trial with a visual perturbation. The red curve shows a typical lateral response to a perturbation. The definition of timing variables is indicated in red. The slope of the green line is the vigor of the response. **(C)** The two types of visual perturbation, each with two amplitudes and two directions.

The setup is identical to that used in previous research in our lab (Zhang et al., 2018). Participants stood in front of a horizontal screen (60 Hz refresh rate, 91.9 × 51.6 cm, 1,920 × 1,080 pixel resolution) lying flat, face-up on a height-adjustable table (Figure 1A). They stood barefoot with their feet separated by about 10% of their height, 15 cm from the near edge of the screen. Table height was adjusted to align the screen with the participant’s hip.

An Optotrak 3,020 motion capture system (Northern Digital, Waterloo, ON, Canada) sampling at 200 Hz was used in the experiment, with a camera located to the right of the participant and another located behind the participant. A photodiode was attached to the far-right corner of the screen to help synchronize the target’s appearance and when the target changed position or the background started to move with the movement measurements (to within 5 ms). The posture was recorded with customized cluster markers: three markers attached rigidly to each other in a triangular configuration. Cluster markers were attached to the forehead, 3rd thoracic vertebra (referred to as “upper trunk”), 1st sacral vertebra (referred to as “lower trunk”) and the wrist (ulnar side). A single marker was attached to the nail of the index finger of the right hand. This marker was used to control the experiment and analyze the movement of the finger.

The timeline of one trial is shown in Figure 1B. A target appeared at a random time between 0.6 s and 1.2 s after the participant placed the right index finger at the starting point. The participant was instructed to tap on the target as accurately and fast as possible with the tip of the right index finger. As soon as the participant started moving towards the target, a visual perturbation (either target jump or background motion) occurred in 80% of the trials. Due to delays in measuring the movement of the finger and rendering images on the screen, the perturbation occurred 60 ms after the finger had moved 5 mm from the starting point. If the target was hit (i.e., if the contact position of the finger was within the target), a sound indicated success. Otherwise, the target drifted away from where the finger touched the screen.

There were nine conditions in 300 fully randomized trials: one condition with no perturbation (60 trials), and eight conditions with a perturbation (30 trials each). The eight conditions resulted from all combinations of two kinds of perturbation (target jump or background motion), two directions (left or right) and two magnitudes (small or big). The checkerboard-like background (square length: 7 cm) was always present (Figure 1C). In the target jump conditions, the target was displaced by either 1 or 4 cm, leftwards or rightwards, across a stationary background. In the background motion conditions, the background moved continuously either leftwards or rightwards at 20 or 60 cm/s, “behind” the stationary target. Before the 300 trials of the experiment, the participants practiced for about 20 trials (random conditions). During the experiment, they could rest at any time between trials by delaying placing their finger at the starting point.

In order to be able to judge whether the two age groups differed in their physical ability to reach while standing, we determined the functional reach ratio (the functional reach distance (Weiner et al., 1992) divided by the individual’s height) before the experiment. Participants stood normally with their feet about shoulder width apart, close to a wall, with the arm that was closest to the wall pointing forward (90° of shoulder flexion). They were instructed to lean forward from this position to reach as far as possible without lifting their heels. A yardstick attached to the wall at the level of the shoulder was used to determine the horizontal distance between the initial and farthest position of the participants’ right fingertip. The maximal reach distance of three trials was considered the functional reach distance.

### Data Analyses

The data analysis was similar to that in our previous study (Zhang et al., 2018), with in addition comparisons involving the two age-groups using two-way analysis of variance (ANOVA) and *t*-tests, and an analysis of the correlation between response latency and movement time.

The 3D kinematic data of all markers were filtered using a second order low-pass Butterworth filter with a cut-off frequency of 30 Hz. We determined this cut-off frequency by determining the minimum variance in the distances between the three markers on a cluster (Schreven et al., 2015). We excluded trials (5%) for which the trial duration or the delay in presenting the perturbation was not within ±3 SD of the mean, or for which the moment of the perturbation could not be determined properly (on the basis of the signal picked up by the photodiode).

#### Dependent Measures

As a measure of accuracy, we defined tapping error as the distance between the endpoint of the movement and the target center. Movement time was determined for each trial as the time from when the finger started moving (finger lifted higher than 5 mm) until it tapped on the screen (i.e., a trial ends). When using movement time as a measure of how fast a participant moved, we averaged the movement time across all nine conditions.

The focus of our study is on the online adjustment to the perturbations that occurred during the movements. As the perturbations were always perpendicular to the main (sagittal) movement direction, we only analyzed the lateral component of the participants’ movements. We did so for the finger, wrist, head, upper trunk and lower trunk. The lateral velocity of the finger was calculated from the measured position data using the central difference algorithm. Responses for each participant were determined by taking the difference in average lateral velocity between trials with a rightward and trials with a leftward perturbation and divided this difference by two. The resulting “lateral response” is positive if it is in the direction of the perturbation. The magnitude of the peak velocity was determined for each age group (young and older) and perturbation type (target jump and background motion) by averaging the peak values of the individual mean responses across participants. These values will be close to the peaks in the lateral response if the timing of the responses is consistent across participants.

The response latency was determined by an extrapolation method: the time at which a line through the points at which the lateral response reached 25% and 75% of the peak response intersected the baseline (no response) value (Figure 1B; Veerman et al., 2008). We use the slope of this line (acceleration) as our measure of the vigor of the response. We defined time zero as the moment at which the perturbations actually happened on the screen. The baseline value was the average response from 50 ms before to 50 ms after this moment.

The extrapolation method requires a clearly identifiable peak. As the lateral response is very modest with respect to the spontaneous trial-to-trial variability for body parts other than the finger, it had multiple peaks for some participants, so it was impossible to reliably identify response peaks for all individual participants. We therefore determined the latencies from the average response of all participants. We bootstrapped (DiCiccio and Efron, 1996) the trials within each participant to obtain a measure of reliability (resampled with replacement). We averaged the resampled responses of all participants and determined the latency for the average response. Doing so 1,000 times provided a distribution of latencies based on resampled trials, which we used to determine a Bayesian 95% credible interval. We performed the data-analysis on all participants. As we used the same data for the young participants as in our previous article, this yielded exactly the same results, except for the results of the bootstrapping which involves a random factor in the resampling.

#### Statistics

Descriptive data are shown as means or means ± SD across participants. As the initial response (and thus the latency) is independent of perturbation amplitude (Zhang et al., 2018), the results are averaged across the two perturbation amplitudes for all analyses except for the plots of the lateral response as a function of time from the perturbation. A 2 × 3 two-way ANOVA was used to test the effects of age (young and older adults; between participants) and perturbation type (no perturbation, target jump and background motion; within participants) on movement time. As we cannot determine a response for the “no perturbation” trials, a similar 2 × 2 ANOVA excluding the “no perturbation” type was used to test the effects of aging and perturbation type on finger response latency. The relationship between response latency and movement time was evaluated with a Pearson correlation. Bayesian 95% credible intervals were determined for the average response latencies across all participants. The tapping error, the accuracy and the functional reach ratio of the young and older groups were compared using *t*-tests. *P* < 0.05 was considered as significant.

## Results

Both age groups performed the task well (success rate above 95%). The average tapping error was similar for both groups across all conditions: 1.46 ± 0.10 cm for the young adults and 1.41 ± 0.07 cm for the older adults. The functional reach ratio was slightly lower in the older group (young: 22.7% ± 3.9%, older: 19.9% ± 3.7%, *p* = 0.043).

The average movement times of the older adults was 526 ± 86 ms, much slower than the 383 ± 44 ms for the young adults (*F*_(1,90)_ = 141.371, *p* < 0.001). The movement time did not depend on the perturbation type (*F*_(2,90)_ = 1.343, *p* = 0.27) and there was no interaction between age and perturbation type (*F*_(2,90)_ = 0.182, *p* = 0.83), so we averaged movement time across all nine conditions of each participant and used this average value for the further analysis.

### Manual, Head and Trunk Responses

**Figure 2:**
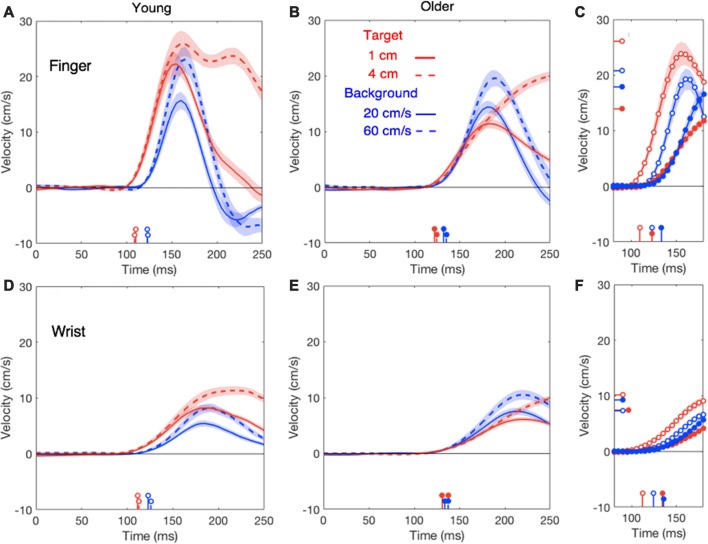
Lateral finger responses (upper panels) and lateral wrist responses (lower panels) in the young **(A,D)** and older **(B,E)** adults as a function of the time after the perturbation. Summary panels on the right show the initial responses of finger **(C)** and wrist **(F)** averaged across the two perturbation sizes for both young adults (open dots) and older adults (filled dots). In these panels, the horizontal lines on the velocity axis show the average of the individual peak responses for each age-group and type of perturbation. In all panels, response onsets are marked by vertical lines on the time-axis. Shaded areas represent the standard error across participants. Data for the young adults are replotted from Zhang et al. (2018).

The first 100 ms of the lateral responses of the finger and wrist were larger for target jumps than for background motion for the young adults, whereas the opposite appears to be the case for the older adults (Figure 2). The difference is mainly due to a much weaker response to target jumps for the older adults (red curves) with a similar response as the young adults for background motion. In general, responses to small and large perturbations had very similar latencies but the larger perturbations gave rise to slightly larger response amplitudes. After averaging the responses to the two perturbation sizes, both for target jumps and for background motion (Figures 2C,F), it is clear that all manual responses are delayed for the older adults. Aging also reduced the vigor of the response, but much less so for background motion than for target jumps. The wrist may even respond more strongly to background motion for the older adults than for the young adults (filled blue dot in Figure 2F is above the open one; also compare blue curves in Figures 2D,E).

**Figure 3:**
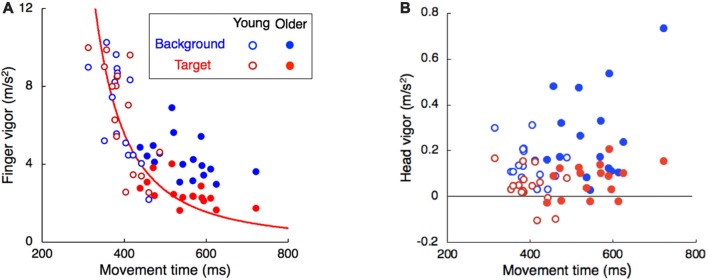
Vigor of the response of the **(A)** finger and **(B)** head as a function of the time between movement onset and tap. Each participant is represented by two dots in each panel, one for the target jump (red) and the other for background motion (blue). The red curve in **(A)** indicates the vigor of a minimal jerk movement adjustment in the time between the onset of the adjustment until the tap. Note that the vigor axis has a different scale in the two panels. The negative values for the vigor in the right panel correspond to participants with head responses in the direction opposite to the target jump.

It is known that the finger responds less vigorously to target jumps when the (remaining) movement time is long (Oostwoud Wijdenes et al., 2011). The vigor of the finger’s response was clearly lower when movement time was longer (red dots in Figure 3A), with the young adults (open symbols) being responsible for the shorter movement times. For responses to a target jump, we can determine the optimal smooth response given the remaining time, considering the delays in the equipment and the average response latency (Flash and Hogan, 1985). The red curve in Figure 3A is the vigor that one would expect for such an optimal response. The overall pattern in the data of both groups (red symbols) is very similar to what one would expect for an optimal smooth response (curve). For the older adults, we see a more vigorous response to background motion than to target jumps (solid blue dots above the red dots). As it is unclear how much one should correct for background perturbations, we cannot make predictions for the vigor of these responses.

**Figure 4:**
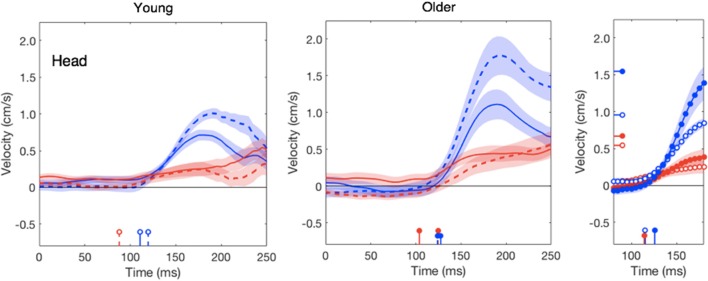
Lateral head responses as a function of the time after the perturbation in the young and older adults. Details as in Figure 2.

**Figure 5:**
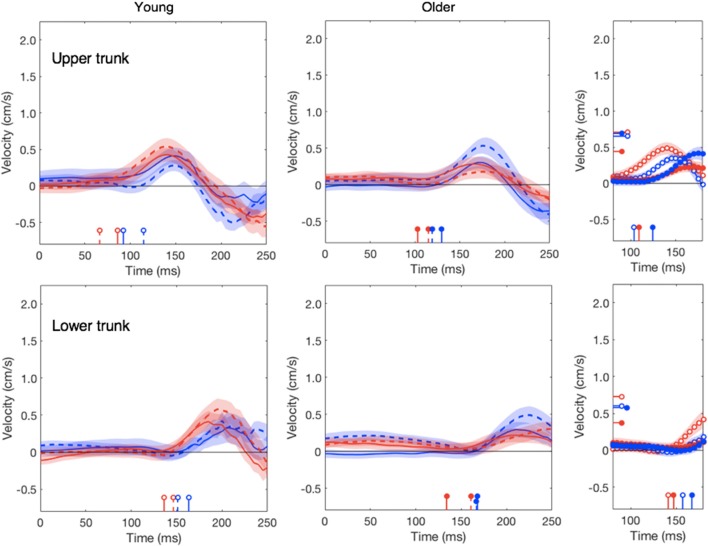
Lateral responses of upper and lower trunk as a function of the time after the perturbation. Details as in Figure 2. In the upper right panel, the latency of the response of the young adults’ upper trunk to a target jump was 66 ms, which is outside the plotted range.

In line with our previous study (Zhang et al., 2018), the head does not respond clearly to target motion; this was independent of the age (red traces in Figure 4). The response to background motion is considerably larger for older than for young adults (compare filled and open blue dots in right panel of Figure 4). Unlike the vigor of finger responses (Figure 3A), the vigor of head responses to background motion does not decrease with movement time (Figure 3B). This is not inconsistent with an explanation in terms of the remaining movement time, as there is no remaining time for the head. The trunk responded to the perturbations in much the same way as the wrist, with older adults having a clearly smaller response to target jumps than young adults, whereas the responses to background motion did not differ (Figure 5).

### Response Latency

**Figure 6:**
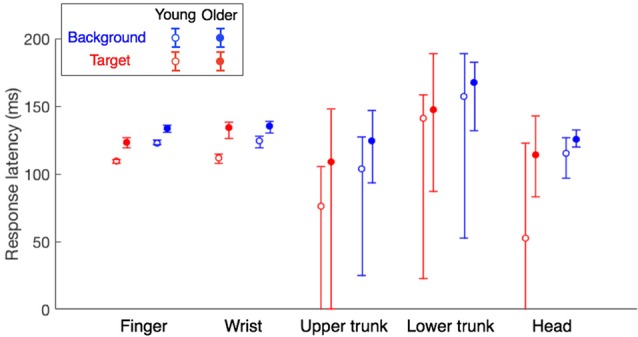
Response latencies of different body parts for the two age groups. Error bars show Bayesian 95% credible intervals that were obtained through bootstrapping (1,000 samples). Data for the young adults are reanalyzed from Zhang et al. (2018).

It is clear that all response latencies were shorter for the young adults than for the older adults (filled symbols higher than open symbols in Figure 6). In line with the results of our previous study (Zhang et al., 2018), the response latency was also shorter for responses to target jumps than for responses to background motion (blue symbols higher than red symbols). For the finger, both the effect of age group and that of perturbation type were significant (*F*_(1,60)_ = 44.6, *p* < 0.001; *F*_(1,60)_ = 42.2, *p* < 0.001) without a significant interaction (*F*_(1,60)_ = 0.81, *p* = 0.37). The same was true for the wrist (age: *F*_(1,60)_ = 44.5, *p* < 0.001; type: *F*_(1,60)_ = 6.57, *p* = 0.013; interaction: *F*_(1,60)_ = 2.89, *p* = 0.094). The latency of the older adults’ finger responses was 126 ± 9 for the target jump and 137 ± 8 for background motion, 11–14 ms later than those of young adults (112 ± 7 and 126 ± 6, respectively). Their wrist responses were 16–22 ms later (Figure 6). A similar trend can be seen for responses of the trunk and head, but it is less clear because of the large variability in the estimated response latencies.

**Figure 7:**
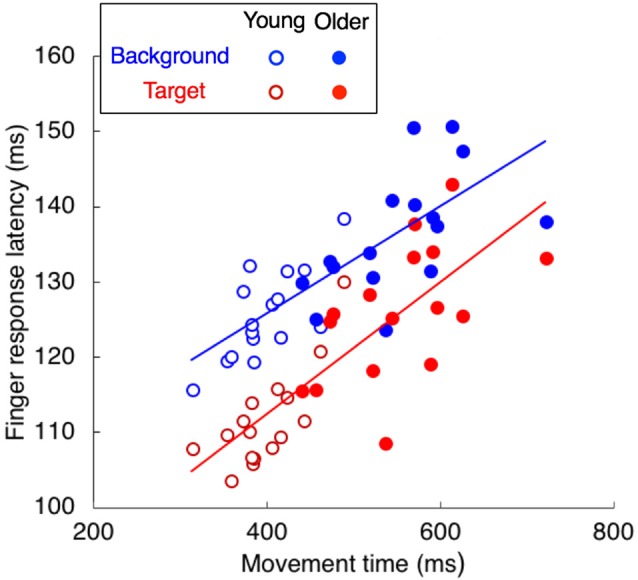
The relationship between finger response latency and movement time. Each participant is represented by two dots, one for the target jump (red) and the other for background motion (blue).

To investigate whether the longer latencies for the older adults could be related to the individual differences in movement time, we plotted the relationship between movement time and finger response latency (Figure 7). The response latency was clearly correlated with the movement time, both for background motion (*r* = 0.783, *p* < 0.001, slope = 0.071) and for target jumps (*r* = 0.811, *p* < 0.001, slope = 0.088), so the longer response latencies for the older adults are in line with their longer movement times.

## Discussion

In this study, we investigated how aging affects the ability to adjust goal-directed movements to sudden visual perturbations (a target jump or background motion). Additionally, we evaluated whether any effects of aging on the adjustments’ timing or vigor could be related to effects on other aspects of movement execution, such as movement time. The patterns of responses to target jumps and background motion were similar to those in our previous study (Zhang et al., 2018). The hand and trunk of young adults responded more vigorously to the target jumps than to background motion, whereas those of the older adults had the opposite pattern of responses (Figures 2, 5). Older adults also had longer movement times and longer response latencies. The increase in response latency with age (about 15 ms) is close to previously reported values of 16–17 ms (Kadota and Gomi, 2010) and 20 ms (Kimura et al., 2015) for fast (~110 ms) responses. A possible explanation for the longer latencies in older adults is sensory slowing. Aging may have negative effects on visual processing speed (Fiorentini et al., 1996; Habekost et al., 2013). An alternative explanation is that the latencies are secondary to a general slowing of movements.

Aging has different effects on the vigor of the various responses. The reduction of vigor with age could be a manifestation of a general slowing process, in which all factors related to force-impulse control could be involved, such as age-related loss of spinal motor neurons and motor units, a decrease in muscle fiber number and cross-sectional area (Booth et al., 1994) and the associated decrease in muscle strength (Goodpaster et al., 2006). We evaluated this by determining the maximal ability in forward reaching without time constraints. As observed in other studies (Duncan et al., 1990; Hageman et al., 1995), the older adults had a slightly lower functional reach ratio. However, as the perturbation was always at the start of the movement, older adults had more time to correct their movement and could therefore use less vigorous responses to achieve an optimally smooth correction (red curve in Figure 3A). Longer movement times could thus be the explanation of the less vigorous finger response to target jumps in older adults. If the reduction of the response vigor with age is related to the remaining time to reach the target, rather than with muscle weakness, we should find very little effect of aging on the responses that are not directly related to reaching the goal. This is indeed the case: the vigor of the finger’s response to background motion did not decrease as much with movement time (and thus age) as that to target jumps (blue dots in Figure 3A), and the vigor of the head responses to target motion even tends to increase with age (red symbols in Figure 3B). A similar pattern can be found in the peak velocities of these responses (right panels of Figures 2, 4).

The increased vigor of the head’s response to background motion for the older adults (Figures 3B, 4) suggests that the elderly rely more on vision to keep their head stable. Several authors have reported that the elderly rely more on vision to control posture (Jamet et al., 2004; Bugnariu and Fung, 2007; Poulain and Giraudet, 2008; Slaboda et al., 2011; Agathos et al., 2015). This could be because the precision of other senses (e.g., vestibular) deteriorates faster with age, or might be caused by the elderly being less good at ignoring irrelevant information (de Dieuleveult et al., 2017). Haibach et al. (2009) found that although sway was more sensitive to the optic flow in older as compared to young adults, in accordance with a higher reliance on vision, the sensation of self-motion (vection) did not increase in parallel. This suggests that the subconscious use of optic flow may become increasingly important with age independently of the explicit perception of self-motion. How the weight given to sensory information changes with age depends on the task. For instance, Wiesmeier et al. (2015) reported that when the task was to maintain balance on a moving platform, the elderly relied to a greater extent on proprioceptive rather than visual and vestibular cues.

If the manual responses to background motion are unnecessary adjustments for moving the hand to the target as a result of assumed self-motion (Gomi, 2008), then the pattern of responses to background motion that we found (Figure 3A) might be a combination of vigor decreasing with increasing movement time in the same way as for target motion, but being larger for the older adults due to an increase in reliance on vision (optic flow) to compensate for sway. If background motion gives rise to compensatory postural adjustments of the hand, head and trunk in order to stabilize the body when confronted with evidence of self-motion (Mergner et al., 2005), the finger’s response to background motion may simply be the result of a misplaced postural correction.

Longer adjustment latencies are clearly related to longer movement times, irrespective of perturbation type (Figure 7). Since the latency of responses to visual perturbations is independent of the remaining movement time (Oostwoud Wijdenes et al., 2011), it is unlikely that the longer latencies in the elderly are a result of the reduced temporal constraints given the longer movement times. On the other hand, the reduced vigor of the finger’s response in the elderly is probably a result of the longer movement time (Figure 3A). Assuming that all participants optimized the combination of speed and accuracy as instructed, the movement time is presumably determined on the basis of the quality of the online control. Thus, most of the age-related differences that we found are probably interrelated, probably with the increased response latency as the origin. Longer latencies in feedback loops lead to unstable behavior unless the gains are low (Burdet et al., 2006), so the corrections are less vigorous in the elderly. The longer movement time is a mechanism for compensating for adjustments being less vigorous and having a longer latency (Salthouse, 1979). With a longer movement time the older adults could perform as accurately as the young adults (though not quite as fast).

In conclusion, our study shows that the general slowing effect of aging includes a longer delay in using visual feedback. The study also confirms that older adults rely more on the visual surrounding to control their movements, and therefore are more affected by background motion. The other effects that we found may be secondary to the increased latency of online adjustments.

## Author Contributions

YZ, EB and JS designed this study. YZ collected all the data and analyzed them with the help of EB and JS. All authors contributed to the interpretation of the data and writing of the manuscript.

## Conflict of Interest Statement

The authors declare that the research was conducted in the absence of any commercial or financial relationships that could be construed as a potential conflict of interest.
